# The Stochastic Evolution of a Protocell: The Gillespie Algorithm in a Dynamically Varying Volume

**DOI:** 10.1155/2012/423627

**Published:** 2012-03-07

**Authors:** T. Carletti, A. Filisetti

**Affiliations:** ^1^Namur Center for Complex Systems and University of Namur, 5000 Namur, Belgium; ^2^CIRI, Energy and Environment Interdipartimental Center for Industrial Research, University of Bologna and European Centre for Living Technology, Cá Foscari University of Venice, 30124 Venice, Italy

## Abstract

We propose an improvement of the Gillespie algorithm allowing us to study the time evolution of an ensemble of chemical reactions occurring in a varying volume, whose growth is directly related to the amount of some specific molecules, belonging to the reactions set. This allows us to study the stochastic evolution of a protocell, whose volume increases because of the production of container molecules. Several protocell models are considered and compared with the deterministic models.

## 1. Introduction

All known life forms are composed of basic units called *cells*; this holds true from the single-cell prokaryote bacterium to the highly sophisticated eukaryotes, whose existence is the result of the coordination, in term of self-organization and emergence, of the behavior of each single basic unit. 

While present-day cells are endowed with highly sophisticated regulatory mechanisms, which represent the outcome of almost four billion-years of evolution, it is generally believed that the first life forms were much simpler. Such primordial life-bricks, the *protocells*, were most probably exhibiting only few simplified functionalities, that required a primitive embodiment structure, a protometabolism, and rudimentary genetics, so to guarantee that offsprings were “similar” to their parents [1–3]. 

Intense research programs are being established aiming at obtaining protocells capable of growth and duplication, endowed with some limited form of genetics [3–6]. Despite all efforts, artificial protocells have not yet been reproduced in laboratory and it is thus extremely important to develop reference models [6–9] that capture the essence of the first protocells appeared on Earth and enable to monitor their subsequent evolution. Due to the uncertainties about the details, high-level abstract models are particularly relevant. Quoting Kaneko [10], it is necessary to “consider simplified models able to capture universal behaviors, without carefully adding complicating details.” 

Most of the models present in the literature are based on deterministic differential equations governing the evolution of the concentrations of the involved reacting molecules. Even if the results are worth discussing and provide important insights, it should be stressed that the former assumptions are rarely satisfied in a cell [11]. Firstly, the number of involved molecules is small and should be counted by integer numbers, hence the use of the concentrations can be questioned; secondly, the presence of the thermal noise introduces in the system a degree of stochasticity than cannot be trivially encoded by a differential equation, mostly because this makes the time evolution a stochastic process. One possible way to overcome such difficulties is to use the Chemical Master equation: given the present state of the system, namely, the number of available molecules for each species, and the possible reactions among them, one can compute the transition probabilities to reach and leave the given state and thus get a partial differential equation describing the time evolution of the probability distribution of having a given number of molecules at any future times [11, 12].

Analytically solving the resulting equation is normally a very hard task, one should thus resort to use numerical methods. A particularly suitable one is the algorithm presented by Gillespie [11, 12], allowing to determine, as a function of the present state of the system, the most probable reaction and the most probable reaction time, that is, the time at which such reaction will occur.

Let us however observe that in the setting we are hereby interested in, the chemical reactions occur in a varying volume, because of the protocell growth; we thus need to adapt the Gillespie method to account for this factor. To the best of our knowledge, there are in the literature very few papers dealing with the Gillespie algorithm in a varying volume [13, 14]. Moreover in all these papers, the volume variation can be considered as an exogenous factor, not being directly related to the number of lipids forming the protocell membrane. So our main contribution is to improve the Gillespie algorithm taking into account the protocell varying volume which is moreover consistent with the increase of the number of lipids constituting the protocell membrane.

The paper is organized as follow. In Section 2 we briefly recall the Surface Reaction Models of protocell that would be used to compare our stochastic numerical scheme. Then in Sections 3 and 4 we will present our implementation of the Gillespie algorithm in a dynamically varying volume. Finally in Section 5 we will present some applications of our method.

## 2. Surface Reaction Models

Among the available models for protocells, a particularly interesting one is the Surface Reactions Model [7, 9], SRM for short, and its applicability to the synchronization problem. Such model is roughly inspired by the Los Alamos bug hypothesis [2, 6] but which, due to its abstraction level, the SRM can be applied to a wider set of protocell hypotheses.

The SRM is built on the assumption that a protocell should comprise at least one kind of “container” molecule (typically a lipid or amphiphile), hereby called *C* molecule, and one kind of replicator molecule—loosely speaking “genetic material,” hereafter called Genetic Memory Molecule, GMM for short, and named with the letter *X*. There are therefore two kinds of reactions which are crucial for the working of the protocell: those which synthesize the container molecules and those which synthesize the GMM replicators,respectively,

(1) $$\begin{array}{c} X_{i}+L_{i}\overset{\alpha_{i}}{\to }X_{i}+C, \end{array}$$

(2) $$\begin{array}{c} X_{i}+P_{j}\overset{M_{ij}}{\to }X_{i}+X_{j}. \end{array}$$

In both cases *L*_*i*_ and *P*_*j*_ are the buffered precursors, respectively, of container molecules and of the *j*th GMM, while *α*_*i*_ and *M*_*ij*_ are the reaction kinetic constants.

A second main assumption of the SRM, is that such reactions occur on the *surface* of the protocell, exposed to the external medium where precursors are free to move. Hence, as long as container molecules are produced, they are incorporated in the membrane that thus increases its size, until a critical point at which, due to physical instabilities, the membrane splits and two offsprings are obtained, each one getting half of the mother's GMMs and whose size is roughly half that of the mother just before the division.

Under the previous assumptions and in the deterministic setting, one can prove [7, 9] that the number of membrane molecules and the number of GMMs evolve in time according to

(3) $$\begin{array}{c} \begin{array}{c} \dot{C}={\left(\frac{C}{\rho }\right)}^{\beta -1}\vec{\alpha }\cdot \vec{X},\\ \dot{\vec{X}}=C^{\beta -1}M\cdot \vec{X}, \end{array} \end{array}$$

where $\vec{X}=(X_{1},\ldots ,X_{N})$ represents the amount of each GMM, $\vec{\alpha }=(\alpha_{1},\ldots ,\alpha_{N})$ is the vector of the reaction constants responsible for the production of *C* molecules from the *X* molecules plus some appropriate precursor. (*M*_*ij*_) denotes the reaction constant at which *X*_*i*_ is produced by *X*_*j*_ plus some precursor. *β* ∈ [2/3,1] is a geometrical shape factor that relates the surface to the volume of the protocell and *ρ* is the lipid density (for more details the interested reader can consult [7, 9]). Let us observe that in this setting the precursors are assumed to be buffered and thus their amount to be constant, hence the latter can be incorporated into the constants *α* and *M*.

So starting with an initial value of container molecules, *C*(*t*_0_) = *C*_0_, and of GMMs, $\vec{X}(t_{0})={\vec{X}}_{0}$, the protocell will grow until some time *t*_0_ + Δ*T*_1_ at which the amount of *C* molecules has doubled with respect to the initial value, *C*(*t*_0_ + Δ*T*_1_) = 2*C*_0_ and thus the protocell undergoes a division. Each offspring will get half of the GMMs the mother protocell had just before the division, ${\vec{X}}^{\left(1\right)}=\vec{X}(t_{0}+\Delta T_{1})/2$. And the protocell cycle starts once again. One can prove [7, 9] that under suitable conditions ${\vec{X}}^{\left(n\right)}$ tends to a constant value once *n* goes to infinity, implying thus the emergence of synchronization of growth and information production.

## 3. The Method

Let us now improve the previous scheme by introducing a probabilistic setting *à la Gillespie*. We thus consider a protocell made by a lipidic vesicle and containing a well-stirred mixture of *N* GMMs, *X*_1_,…, *X*_*N*_, that may react through *m* elementary reaction channels *R*_*μ*_, *μ* = 1,…, *m*, running within the volume *V*(*t*) of the protocell.

Let us observe that because of the protocell growth the volume is an increasing function of time. Actually one can relate the volume to the amount of container molecules via their density *V* = *C*/*ρ* where *C* denotes the integer number of molecules forming the lipidic membrane. We will hereby use the same symbol *X*_*i*_ to denote both the *i*th GMM and the integer number of molecules of type *X*_*i*_ in the system.

For each reaction channel *R*_*μ*_ assume that there exists a scalar rate *c*_*μ*_ such that *c*_*μ*_ *dt* + *o*(*dt*) is the probability that a random combination of molecules from channel *R*_*μ*_ will react in the interval [*t*, *t* + *dt*) within the volume *V*(*t*).

Let *h*_*μ*_(*Y*) be the total number of possible distinct combinations of molecules for a channel *R*_*μ*_ when the system is in state *Y* = (*X*_1_,…, *X*_*N*_, *C*), then we can define the *propensity* [14] of the reaction *R*_*μ*_ to be *a*_*μ*_(*Y*) = *h*_*μ*_(*Y*)*c*_*μ*_.

One can prove [11] that for a binary reaction the rate *c*_*μ*_ can be written in the form *c*_*μ*_ = *k*_*μ*_/*V*, where *k*_*μ*_ is a fixed constant. Similarly one can prove that for a reaction involving *n* different species, we get *c*_*μ*_ = *k*_*μ*_/*V* ^*n*−1^. And thus for a single molecule reaction, that is, a decay, we get *c*_*μ*_ = *k*_*μ*_, namely, independently from the volume.

Let us now assume that among the *m* reactions, *Q*_1_ involve one single molecule, *Q*_2_ are binary reactions, *Q*_3_ are ternary reactions, and so on. Of course *Q*_1_ + *Q*_2_ + ⋯+*Q*_*N*+1_ = *m*. We recall that we have *N* GMMs and the container-type molecule *C*, hence *N* + 1 species. For short we will denote 𝒬_1_ the set of indices *μ* for monomolecule reactions, and by 𝒬 the remaining ones. Let us observe that in this way some coefficient *a*_*μ*_, will depend both on the system state *Y* and on the time via the volume *V*(*t*): *a*_*μ*_(*Y*, *t*) for *μ* ∈ 𝒬.

More precisely to study the time evolution of the system we need to determine the probability *P*_*μ*_(*τ* | *Y*, *t*)*dτ*, that given the system in the state *Y* = (*X*_1_,…, *X*_*n*_, *C*) at time *t*, then the next reaction will occur in the infinitesimal time interval (*t* + *τ*, *t* + *τ* + *dτ*) and it will be the reaction *R*_*μ*_. This probability will be computed as

(4) $$\begin{array}{c} P_{\mu }\left(\tau \;|\;Y,t\right)d\tau =P_{\text{not}}\left(\tau \;|\;Y,t\right)\times a_{\mu }\left(Y,t+\tau \right)d\tau , \end{array}$$

where *P*_not_(*τ* | *Y*, *t*) is the probability that no reaction occurs in (*t*, *t* + *τ*) given the state *Y* at time *t* whereas the rightmost term denotes the probability to have a reaction *R*_*μ*_ in (*t* + *τ*, *t* + *τ* + *dτ*) given the state *Y* at time *t* + *τ*.

To compute the first term *P*_not_, let us take *s* ∈ [*t*, *t* + *τ*] and observe that:

(5) $$\begin{array}{cc} P_{\text{not}}\left(s+ds\;|\;Y,t\right) & =P_{\text{not}}\left(s\;|\;Y,t\right)P_{\text{not}}\left(ds\;|\;Y,t+s\right)\\ & {=P}_{\text{not}}\left(s\;|\;Y,t\right)\left(1-\underset{\mu }{\sum }a_{\mu }\left(Y,t+s\right)ds\right), \end{array}$$

being 1 − ∑_*μ*_ *a*_*μ*_(*Y*, *t* + *s*)*ds* the probability that no reaction will occur in (*t* + *s*, *t* + *s* + *ds*) once we are in state *Y* at time *t* + *s*. Thus rewriting the previous difference equation as a differential equation, passing to the limit *ds* → 0, and observing that *P*_not_(0 | *Y*, *t*) = 1, we get the solution:

(6) $$\begin{array}{c} P_{\text{not}}\left(\tau \;|\;Y,t\right)=\exp\left[-A_{Q_{1}}\left(Y\right)\tau -\overset{\tau }{\underset{0}{\int }}A_{Q}\left(Y,s+t\right)ds\right], \end{array}$$

where

(7) $$\begin{array}{c} A_{Q_{1}}\left(Y\right)=\underset{\mu \in {𝒬}_{1}}{\sum }a_{\mu }\left(Y\right),\;\;\;A_{Q}\left(Y,s+t\right)=\underset{\mu \in 𝒬}{\sum }a_{\mu }\left(Y,s+t\right). \end{array}$$

The apparent asymmetry in the exponential term in (6) is easily recovered by observing that *A*_*Q*_1__(*Y*)*τ* = ∫_0_^*τ*^ *A*_*Q*_1__(*Y*)*ds*.

We can thus conclude that

(8) $$\begin{array}{cc} & P_{\mu }\left(\tau \;|\;Y,t\right)d\tau \\ & \;=\exp\left[-A_{Q_{1}}\left(Y\right)\tau -\overset{t+\tau }{\underset{t}{\int }}A_{Q}\left(Y,s\right)ds\right]a_{\mu }\left(Y,t+\tau \right)d\tau . \end{array}$$

Let us observe that the rightmost term is correctly *a*_*μ*_(*Y*, *t* + *τ*), namely the system is still in the state *Y* at time *t* + *τ*, because no reaction has been produced in (*t*, *t* + *τ*).

Let us recall that the volume enters in the previous relation via the function *A*_*Q*_, more explicitly one has

(9) $$\begin{array}{cc} & A_{Q}\left(Y,s\right)=\underset{\mu \in {𝒬}_{2}}{\sum }\frac{h_{\mu }\left(Y\right)k_{\mu }}{V\left(s\right)}+\underset{\mu \in {𝒬}_{3}}{\sum }\frac{h_{\mu }\left(Y\right)k_{\mu }}{{\left(V\left(s\right)\right)}^{2}}\\ & \;\;\;\;\;\;+\cdots +\underset{\mu \in {𝒬}_{N+1}}{\sum }\frac{h_{\mu }\left(Y\right)k_{\mu }}{{\left(V\left(s\right)\right)}^{N}}, \end{array}$$

that can be rewritten in terms of *C* molecules using the relation *C* = *ρV*. So our method applies to a different problem with respect to the one considered in [14], in fact in our case the volume growth is not imposed a priori but dynamically evolves according to the reaction scheme, if *C* is produced then *V* increases otherwise it will keep a constant value, while in [14] the volume growth is an exogenous variable.

## 4. The Stochastic Simulation Algorithm in a Growing Volume

Once we have the probability function *P*_*μ*_(*τ* | *Y*, *t*) we can build an algorithm that reproduces the time evolution given by the model defined above.

Given the system in some state *Y* at time *t*, we must determine the interval of time *τ* and the reaction channel *R*_*μ*_ according to the probability distribution function *P*_*μ*_(*τ* | *Y*, *t*), and finally update the state *Y* → *Y* + *ν*_*μ*_, where *ν*_*μ*_ is a stoichiometric vector representing the increase and decrease of molecular abundance due to the reaction *R*_*μ*_. This will be accomplished following the standard approach by Gillespie [11] but taking care of the time dependence of the propensities. We will thus need to compute the cumulative probability distribution function and then make use of the inversion method [12], to determine the channel *μ* and the next reaction time *τ*, distributed according to *P*_*μ*_(*τ* | *Y*, *t*).

From (8) we can compute the *cumulative distribution function*

(10) $$\begin{array}{c} F\left(\tau \;|\;Y,t\right)=\overset{\tau }{\underset{0}{\int }}\underset{\mu }{\sum }P_{\mu }\left(s\;|\;Y,t\right)ds, \end{array}$$

providing the probability that any reaction will occur in (*t*, *t* + *τ*) starting from the state *Y* at time *t*. The function *F*(*τ* | *Y*, *t*) can be explicitly computed by the following.

**Proposition 1**

Under the above assumptions we have

(11) $$\begin{array}{c} F\left(\tau \;|\;Y,t\right)=1-\exp\left[-A_{Q_{1}}\left(Y\right)\tau -\overset{t+\tau }{\underset{t}{\int }}A_{Q}\left(Y,s\right)ds\right]. \end{array}$$

**Proof**

The first step is to use (8) and perform a sum over all the channels *μ* to rewrite (10) as

(12) $$\begin{array}{cc} F\left(\tau \;|\;Y,t\right) & =\overset{\tau }{\underset{0}{\int }}\left(A_{Q_{1}}\left(Y\right)+A_{Q}\left(Y,t+s\right)\right)\\ & \;\times \exp\left[-A_{Q_{1}}\left(Y\right)s-\overset{t+s}{\underset{t}{\int }}A_{Q}\left(Y,r\right)dr\right]ds. \end{array}$$

Then we can observe that

(13) $$\begin{array}{cc} & \;\frac{\partial }{\partial s}\left(\exp\left[-A_{Q_{1}}\left(Y\right)s-\overset{t+s}{\underset{t}{\int }}A_{Q}\left(Y,r\right)dr\right]\right)\\ & \;\;=-\left(A_{Q_{1}}\left(Y\right)+A_{Q}\left(Y,t+s\right)\right)\\ & \;\;\;\times \exp\left[-A_{Q_{1}}\left(Y\right)s-\overset{t+s}{\underset{t}{\int }}A_{Q}\left(Y,r\right)dr\right], \end{array}$$

and thus

(14) $$\begin{array}{cc} & F\left(\tau \;|\;Y,t\right)\\ & \;=-\overset{\tau }{\underset{0}{\int }}\frac{\partial }{\partial s}\left(\exp\left[-A_{Q_{1}}\left(Y\right)s-\overset{t+s}{\underset{t}{\int }}A_{Q}\left(Y,r\right)dr\right]\right)ds\\ & \;=1-\exp\left[-A_{Q_{1}}\left(Y\right)\tau -\overset{t+\tau }{\underset{t}{\int }}A_{Q}\left(Y,r\right)dr\right]. \end{array}$$

Once we have the cumulative distribution function we can obtain the value *τ* by drawing a random number *u*_1_ from an uniform distribution in [0,1] and then solve with respect to *τ* the implicit equation:

(15) $$\begin{array}{c} u_{1}=1-\exp\left[-A_{Q_{1}}\left(Y\right)\tau -\overset{t+\tau }{\underset{t}{\int }}A_{Q}\left(Y,s\right)ds\right]. \end{array}$$

Let us stress once again that this is not as straightforward as for the original Gillespie [11] scheme, or the simplified one presented in [14], because of the time dependence of *A*_*Q*_ via the volume. One can nevertheless find suitable approximation for the integral, this will be the goal of the next sections.

### 4.1. The Adiabatic Assumption

Let us assume that *τ* is very small, or which is equivalent, that the time scale of the chemical reactions involving the GMMs is much faster than the production of container molecules, hence the volume growth is very slow compared with the production of the chemicals *X*_*i*_.

Under this hypothesis one can assume that in the interval (*t*, *t* + *τ*) the volume does not vary and thus one can make the following approximation

(16) $$\begin{array}{c} \overset{t+\tau }{\underset{t}{\int }}A_{Q}\left(Y,s\right)\;ds\sim A_{Q}\left(Y,t\right)\tau . \end{array}$$

One can thus explicitely solve (15) to get

(17) $$\begin{array}{c} \tau_{\text{Gill}}=-\frac{1}{A_{Q_{1}}\left(Y\right)+A_{Q}\left(Y,t\right)}\log\left(1-u_{1}\right), \end{array}$$

that is the standard Gillespie result except now that *A*_*Q*_(*Y*, *t*) depends on time and as long the volume increases, then the contribution arising from *A*_*Q*_(*Y*, *t*) might become smaller because *A*_*Q*_ ~ 1/*V*.

### 4.2. The Next Order Correction

One can obtain a somehow better estimate valid in the case of comparable time scales for the reactions involving GMM and the container growth. The idea is to compute the integral in (15) using the following approximation:

(18) $$\begin{array}{cc} \overset{t+\tau }{\underset{t}{\int }}A_{Q}\left(Y,s\right)ds & =\overset{\tau }{\underset{0}{\int }}A_{Q}\left(Y,t+s\right)ds\\ & =\overset{\tau }{\underset{0}{\int }}\left(A_{Q}\left(Y,t\right)+\frac{\partial A_{Q}\left(Y,t\right)}{\partial t}s+\cdots \right)ds\\ & =A_{Q}\left(Y,t\right)\tau +\frac{\partial A_{Q}\left(Y,t\right)}{\partial t}\frac{\tau^{2}}{2}+𝒪\left(\tau^{3}\right), \end{array}$$

where ∂*A*_*Q*_(*Y*, *t*)/∂*t* can be obtained using the definition (9) and expressing the volume in terms of *C* = *V*(*t*)*ρ*, namely,

(19) $$\begin{array}{cc} & \frac{\partial A_{Q}\left(Y,t\right)}{\partial t}\\ & \;=-\frac{\dot{C}}{C}(\underset{\mu \in {𝒬}_{2}}{\sum }\frac{h_{\mu }\left(Y\right)k_{\mu }}{C\left(t\right)}+2\underset{\mu \in {𝒬}_{3}}{\sum }\frac{h_{\mu }\left(Y\right)k_{\mu }}{{\left(C\left(t\right)\right)}^{2}}\\ & \;\;\;\;\;\;+\ldots +N\underset{\mu \in {𝒬}_{N+1}}{\sum }\frac{h_{\mu }\left(Y\right)k_{\mu }}{{\left(C\left(t\right)\right)}^{N}}). \end{array}$$

To compute $\dot{C}/C$ we make the assumption that in a very short time interval, as the one we are interested in, the deterministic growth of the container is a good approximation for the stochastic underlying mechanism; this implies that we can use  (3)

(20) $$\begin{array}{c} \frac{\dot{C}}{C}={\left(\frac{C(t)}{\rho }\right)}^{\beta -1}\frac{\vec{\alpha }\cdot \vec{X}\left(t\right)}{C\left(t\right)}. \end{array}$$

Inserting the previous result into (18) and finally solving (15) with respect to *τ*, we can compute the next reaction time up to correction of the order of *τ* ^3^, as follows

(21) $$\begin{array}{cc} \tau_{\text{Gill}} & =\frac{-\left(A_{Q_{1}}\left(Y\right)+A_{Q}\left(Y,t\right)\right)}{{\dot{A}}_{Q}\left(Y,t\right)}\\ & \;+\frac{\sqrt{{\left(A_{Q_{1}}\left(Y\right)+A_{Q}\left(Y,t\right)\right)}^{2}-2\log\left(1-u_{1}\right){\dot{A}}_{Q}\left(Y,t\right)}}{{\dot{A}}_{Q}\left(Y,t\right)}, \end{array}$$

where we wrote for short ${\dot{A}}_{Q}(Y,t)=\partial A_{Q}\left(Y,t\right)/\partial t$ and we selected the positive square root in such a way in the limit ${\dot{A}}_{Q}(Y,t)\to 0$ we recover the previous solution (17).

**Remark 2 (On the existence of *τ*_Gill_)**

In the case of variable volume a new phenomenon can arise: the volume growth can be so fast that no reaction can occur in the interval (*t*, *t* + *τ* + *dτ*) for any *τ*. Mathematically this translates into a sign condition for the term under square root in (21), if

(22) $$\begin{array}{c} \log\left(1-u_{1}\right)<\frac{{\left(A_{Q_{1}}\left(Y\right)+A_{Q}\left(Y,t\right)\right)}^{2}}{\left(2{\dot{A}}_{Q}\left(Y,t\right)\right)}, \end{array}$$

then (15) has no real solution.

**Figure 1:**
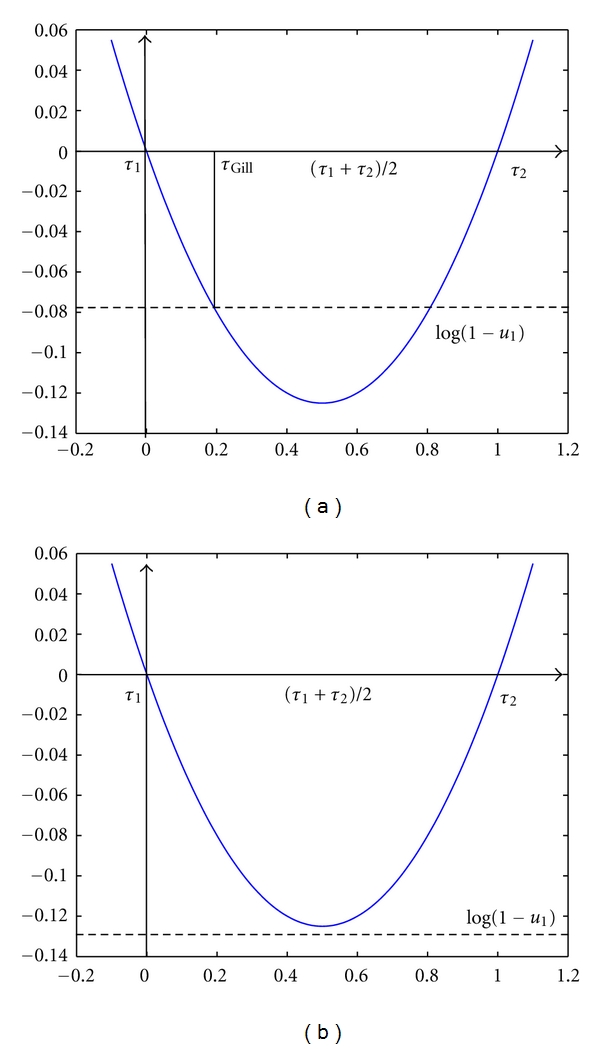
Geometrical interpretation of the existence of the next reaction time *τ*_Gill_. (a) *τ*_Gill_ is the smallest intersection between the parabola and the horizontal line log⁡(1 − *u*_1_). (b) *τ*_Gill_ does not exist, the horizontal line is located below the minimum of the parabola.

This can be geometrically interpreted as follows. The relation (15) determines *τ*_Gill_ as the intersection of the parabola $-A_{Q_{1}}(Y)-A_{Q}(Y,t)\tau -{\dot{A}}_{Q}(Y,t)\tau^{2}/2$ with the horizontal line log⁡(1 − *u*_1_), which is negative because *u*_1_ ∈ (0,1). Such parabola intersect the *y*-axis at *τ*_1_ = 0 and $\tau_{2}=-2(A_{Q_{1}}(Y)+A_{Q}(Y,t))/{\dot{A}}_{Q}(Y,t)>0$ and it is concave. Then its absolute (negative) minimum is reached at the vertex *τ*_*V*_ = (*t*_1_ + *t*_2_)/2 and its value is $\left(A_{Q_{1}}(Y)+A_{Q}{\left(Y,t)\right)}^{2}/(2{\dot{A}}_{Q}(Y,t)\right)$ and it is negative because ${\dot{A}}_{Q}(Y,t)$ is negative. Hence if the horizontal line is below this value, that is, condition (22) is verified, the parabola and the line do not have any real intersections (see Figure 1).

Let us also observe that, whenever it exists, *τ*_Gill_ is always positive as it should be. In the case of a protocell the nonexistence of such next reaction time could be translated into the death by dilution of the protocell.

### 4.3. The Next Reaction Channel

Whenever the next reaction time does exist, the next reaction channel is determined using the classical Gillespie method, namely, by drawing a second uniformly distributed random number *u*_2_ ∈ [0,1] and fix the channel *μ* such that

(23) $$\begin{array}{c} \overset{\mu -1}{\underset{\nu =1}{\sum }}a_{\nu }\left(Y,t+\tau \right)\leq u_{2}a_{0}\left(Y,t+\tau \right)\leq \overset{\mu }{\underset{\nu =1}{\sum }}a_{\nu }\left(Y,t+\tau \right), \end{array}$$

where *a*_0_(*Y*, *t* + *τ*) = *A*_*Q*_1__(*Y*) + *A*_*Q*_(*Y*, *t* + *τ*) = ∑_*ν*=1_^*m*^ *a*_*ν*_(*Y*, *t* + *τ*).

**Remark 3**

Let us observe that if all the reactions involve the same number of chemicals, then the determination of which reaction channel *μ* will be activated in the next reaction does not depend on the volume which factorizes out from (23). In fact assuming all the reactions to involve *p* chemical, we obtain by definition

(24) $$\begin{array}{c} a_{\nu }\left(Y,t+\tau \right)=\frac{h_{\nu }\left(Y\right)k_{\nu }}{{\left[V(t+\tau )\right]}^{p}}\;\forall \nu \in \left\{1,\ldots ,m\right\}, \end{array}$$

and thus (23) rewrites

(25) $$\begin{array}{c} \overset{\mu -1}{\underset{\nu =1}{\sum }}\frac{h_{\nu }\left(Y\right)k_{\nu }}{{\left[V(t+\tau )\right]}^{p}}\leq u_{2}\overset{m}{\underset{\nu =1}{\sum }}\frac{h_{\nu }\left(Y\right)k_{\nu }}{{\left[V(t+\tau )\right]}^{p}}\leq \overset{\mu }{\underset{\nu =1}{\sum }}\frac{h_{\nu }\left(Y\right)k_{\nu }}{{\left[V(t+\tau )\right]}^{p}}, \end{array}$$

which is clearly independent of the volume value *V*.

## 5. Some Applications

The aim of this section is to provide some applications of the previous algorithm to the study of the evolution of a protocell.

### 5.1. One Single Genetic Memory Molecule

The simplest model is the one where only one GMM specie is present in the protocell [9] and thus only two chemical channels are active:

(26) $$\begin{array}{cc} & \text{channel}\;1,R_{1}:\;X+P_{1}\overset{\eta }{\to }2X,\\ & \text{channel}\;2,R_{2}:\;X+L_{1}\overset{\alpha }{\to }X+C, \end{array}$$

where *P*_1_ and *L*_1_ are, respectively, precursors of GMM, that is, nucleotide, and precursors of amphiphiles.

One can thus compute the propensities in the state *Y* = (*X*, *C*) at time *t*:

(27) $$\begin{array}{c} a_{1}\left(X,C,t\right)=h_{1}\left(X,C\right)\frac{\eta }{V\left(t\right)}=\eta \frac{P_{1}X}{V\left(t\right)},\;\\ a_{2}\left(X,C,t\right)=h_{2}\left(X,C\right)\frac{\alpha }{V\left(t\right)}=\alpha \frac{L_{1}X}{V\left(t\right)}, \end{array}$$

let us observe that we assume that precursors are buffered and thus they are constant.

Because system (26) contains only bimolecular reactions, all the propensities are time dependent, hence *A*_*Q*_1__ = 0 and *A*_*Q*_ = *a*_1_(*X*, *C*, *t*) + *a*_2_(*X*, *C*, *t*) = (*P*_1_ *η* + *L*_1_ *α*)*X*/*V*(*t*), thus (15) simplifies into

(28) $$\begin{array}{c} u_{1}=1-\exp\left[-\overset{t+\tau }{\underset{t}{\int }}A_{Q}\left(Y,s\right)ds\right], \end{array}$$

whose second-order solution (21) is given by

(29) $$\begin{array}{cc} \tau_{\text{Gill}} & =\frac{-A_{Q}\left(\text{Y},t\right)}{{\dot{A}}_{Q}\left(Y,t\right)}\\ & \;+\frac{\sqrt{{\left(A_{Q}\left(Y,t\right)\right)}^{2}-2\log\left(1-u_{1}\right){\dot{A}}_{Q}\left(Y,t\right)}}{{\dot{A}}_{Q}\left(Y,t\right)},\\ \frac{\partial A_{Q}\left(X,C,t\right)}{\partial t} & ={-\frac{\dot{V}\left(t\right)}{V\left(t\right)}\left(\frac{P_{1}\eta X}{V\left(t\right)}+\frac{L_{1}\alpha X}{V\left(t\right)}\right)|}_{V(t)=C(t)/\rho }\\ & =-{\left(\frac{C}{\rho }\right)}^{\beta -1}\frac{\rho L_{1}\alpha X^{2}}{C^{2}}\left(P_{1}\eta +L_{1}\alpha \right). \end{array}$$

So we can finally obtain

(30) $$\begin{array}{cc} & \tau_{\text{Gill}}\\ & \;=\frac{C}{L_{1}\alpha X}{\left(\frac{\rho }{C}\right)}^{\beta -1}\\ & \;\;-\sqrt{{\left[\frac{C}{L_{1}\alpha X}{\left(\frac{\rho }{C}\right)}^{\beta -1}\right]}^{2}+2\frac{C^{2}}{L_{1}\alpha \rho X^{2}\left(P_{1}\eta +L_{1}\alpha \right)}\log\left(1-u_{1}\right),} \end{array}$$

provided

(31) $$\begin{array}{c} \log\left(1-u_{1}\right)\geq -\frac{\rho }{2\alpha }{\left(\frac{\rho }{c}\right)}^{2(\beta -1)}\left(P_{1}\eta +L_{1}\alpha \right). \end{array}$$

Which reaction channel *μ* will be active in the time interval [*t*, *t* + *τ*] can be obtained according to

(32) $$\begin{array}{cc} & \text{if}\;\;u_{2}\frac{\left(P_{1}\eta +L_{1}\alpha \right)X}{V}\leq \frac{P_{1}\eta X}{V}\\ & \;\text{namely}\;0\leq u_{2}\leq \frac{P_{1}\eta }{P_{1}\eta +L_{1}\alpha }\;\text{then}\;\mu =1\\ & \text{if}\;\;\frac{P_{1}\eta X}{V}<u_{2}\frac{\left(P_{1}\eta +L_{1}\alpha \right)X}{V}\leq \frac{\left(P_{1}\eta +L_{1}\alpha \right)X}{V}\\ & \;\text{namely}\;\frac{P_{1}\eta }{P_{1}\eta +L_{1}\alpha }<u_{2}\leq 1\;\text{then}\;\mu =2. \end{array}$$

Let us observe that according to Remark 3, the choice of *μ* does not depend on the volume, because only binary reactions are present.

Let *C*_0_ be the initial amount of container molecules, then we assume that once $C(\bar{t})=2C_{0}$ the protocell splits into two offspring, almost halving the GMM amount. More precisely we assume that the first offspring will get a number of GMMs drawn according to a binomial distribution with parameter *p* = 1/2 and $n=X(\bar{t})$. From this step, for technical reason, only one randomly chosen offspring will be studied during each generation.

**Figure 2:**
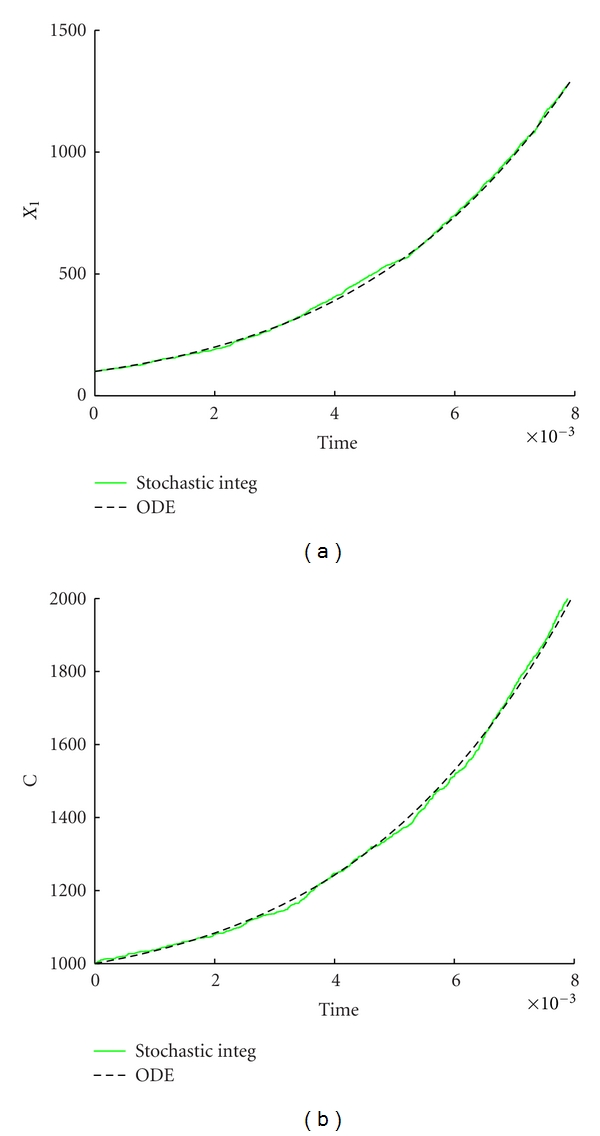
Stochastic versus ODE SRM protocell (3). Case of one GMM, (a) the time evolution of the amount of GMM, (b) the time evolution of the amount of *C*. Parameters are *η* = 1, *α* = 1, *L*1 = 500, *P*1 = 600, *X*_1_(0) = 100, *C*(0) = 1000, *ρ* = 200, and *β* = 2/3.

In Figure 2 we report a comparison between the deterministic (3) and the stochastic dynamics, under the adiabatic assumption for *τ*_Gill_, corresponding to the continuous growth phase of the container between two successive divisions. As one should expect, a system composed by a large number of molecules exhibits small stochastic fluctuations whose average is not too far from the dynamics described by the deterministic model.

**Figure 3:**
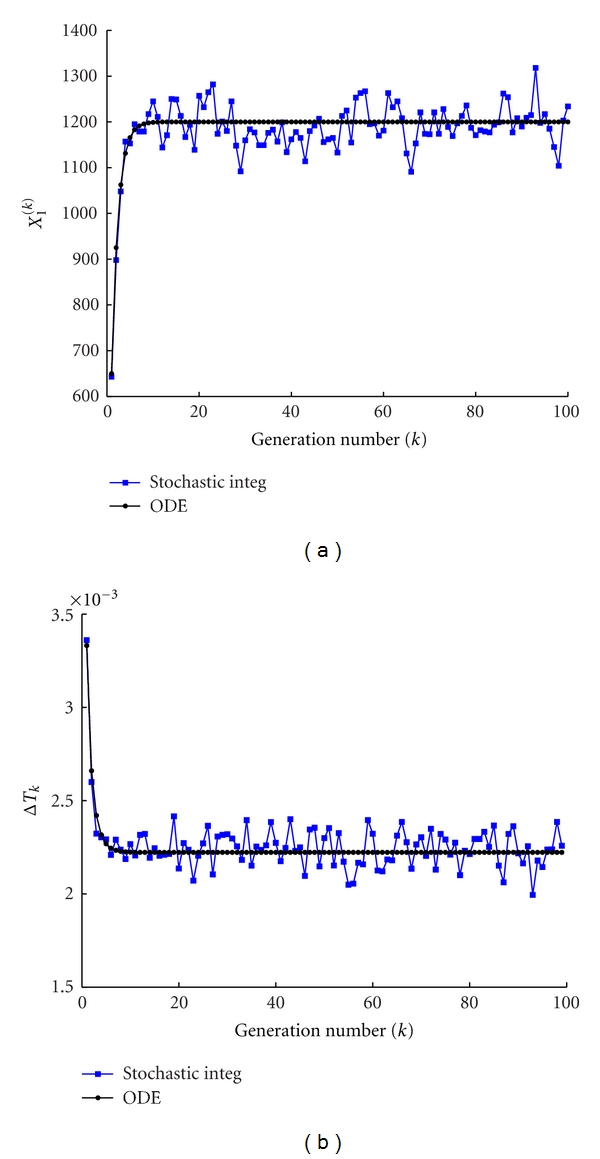
Stochastic versus ODE SRM protocell (3). Case of one GMM, (a) the amount of GMM at the beginning of each division cycle, (b) the division time as a function of the number of elapsed divisions. Parameters are *η* = 1, *α* = 1, *L*1 = 500, *P*1 = 600, *X*_1_(0) = 100, *C*(0) = 1000, *ρ* = 200, and *β* = 2/3.

In Figure 3 we report the amount of GMM, *X* ^(*k*)^ (Figure 3(a)), at the beginning of each protocell cycle and the duplication time (Figure 3(b)), namely, the interval of time needed to double the amount of *C* molecules, for both the stochastic and deterministic models. Once again one can clearly observe the small fluctuations of the stochastic system around the value obtained by the numerical integration of the deterministic description (3). Let us observe that these fluctuations are due to the stochastic integrator scheme and also on the division mechanism.

**Figure 4:**
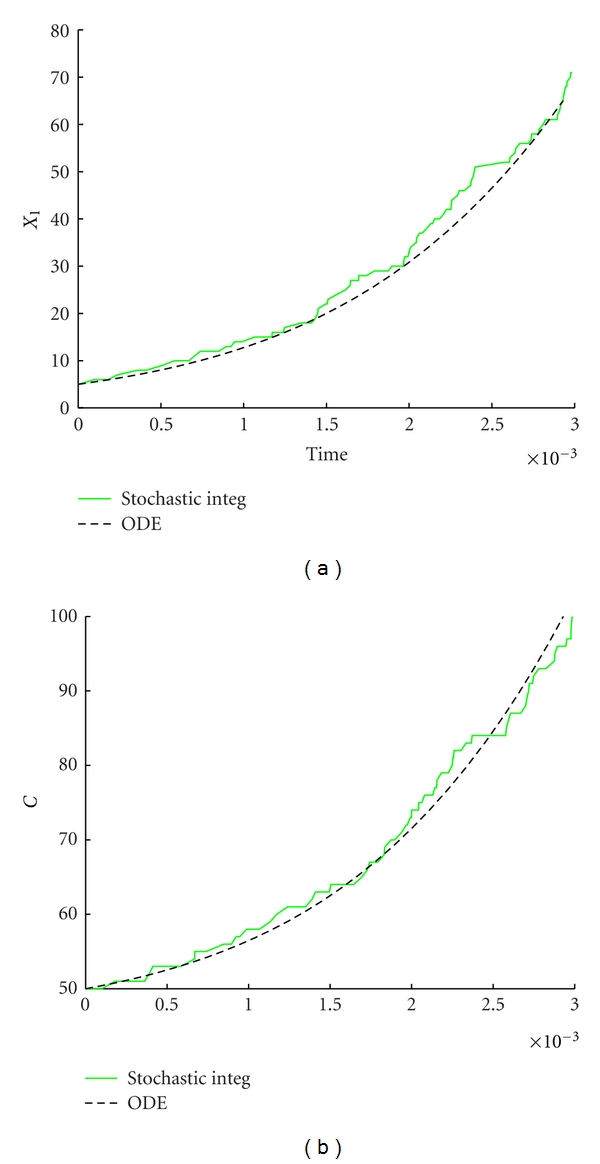
Stochastic versus ODE SRM protocell (3). Case of one GMM, (a) the time evolution of the amount of GMM, (b) the time evolution of the amount of *C*. Parameters are *η* = 1, *α* = 1, *L*1 = 500, *P*1 = 600, *X*_1_(0) = 5, *C*(0) = 50, *ρ* = 200, and *β* = 2/3.

**Figure 5:**
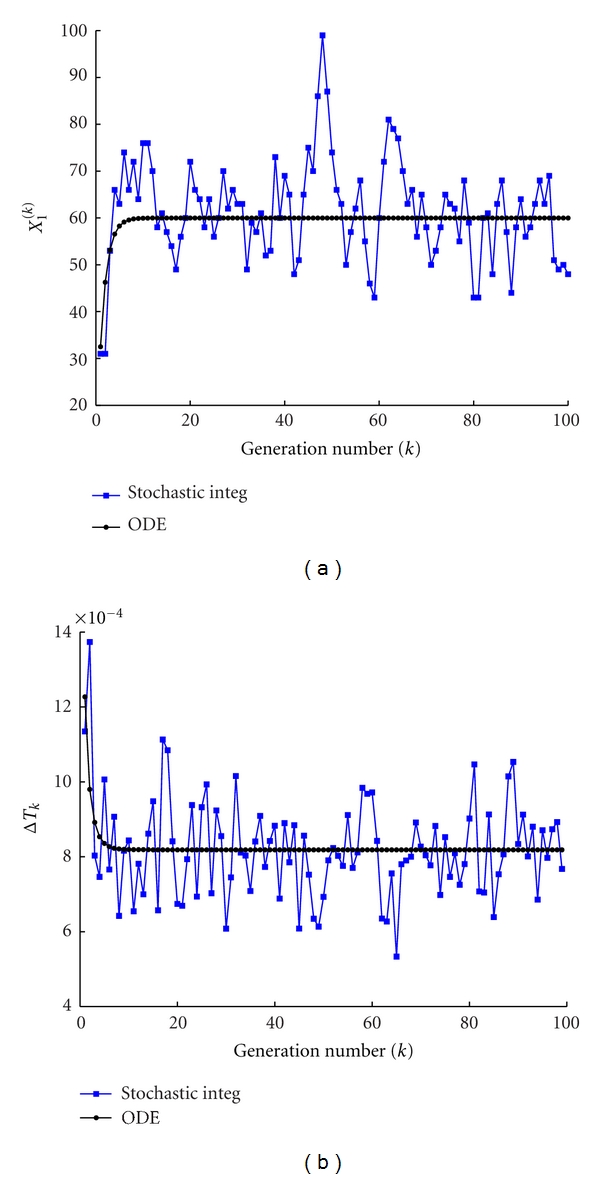
Stochastic versus ODE SRM protocell (3). Case of one GMM, (a) the amount of GMM at the beginning of each division cycle, (b) the division time as a function of the number of elapsed divisions. Parameters are *η* = 1, *α* = 1, *L*1 = 500, *P*1 = 600, *X*_1_(0) = 5, *C*(0) = 50, *ρ* = 200, and *β* = 2/3.

We are now interested in studying the fluctuations dependence on the amount of molecules. We already know that for a sufficiently large number of molecules the stochastic dynamics follows closely the deterministic one and thus the fluctuations are small. On the other hand, one should expect that when the number of molecules decreases, then the fluctuation will rise and the system behavior could not be completely described by means of a deterministic approach. This is confirmed by Figures 4 and 5, where we can observe that a model composed by a small number of initial molecules, 20-times lesser than in the model presented in Figure 2 exhibits larger stochastic fluctuations.

**Figure 6:**
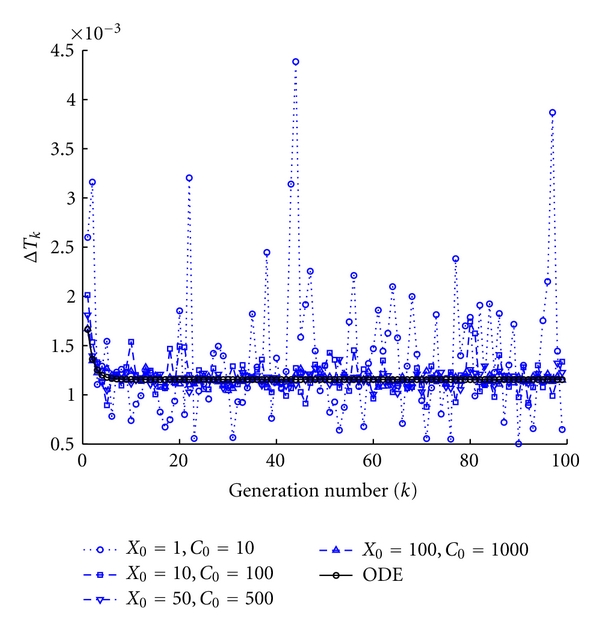
Fluctuation dependence on the initial conditions. We report the division times as a function of the number of elapsed divisions, for 5 different protocells models. Protocell ∘: *X*_1_(0) = 5, *C*(0) = 10, protocell □: *X*_1_(0) = 10, *C*(0) = 100, protocell ∇: *X*_1_(0) = 50, *C*(0) = 500, protocell ∆: *X*_1_(0) = 100, *C*(0) = 1000. The black line denotes the deterministic protocell. All the remaining parameters have been fixed to *η* = 1, *α* = 1, *L*1 = 500, *P*1 = 600, *ρ* = 100, and *β* = 1.

In Figure 6 we summarize the results of several protocell models each one with a different amount of initial molecules, in order to appreciate the influence of the latter on the stochastic fluctuations. To compare with, we also report the case of the deterministic model. Because the kinetic constants are kept constant, the analytical theory for the deterministic model ensures that the division time does not vary [7]. Nevertheless the fewer the initial amount of *X*_0_ and *C*_0_ is, the larger the fluctuations present in the stochastic integration are.

**Figure 7:**
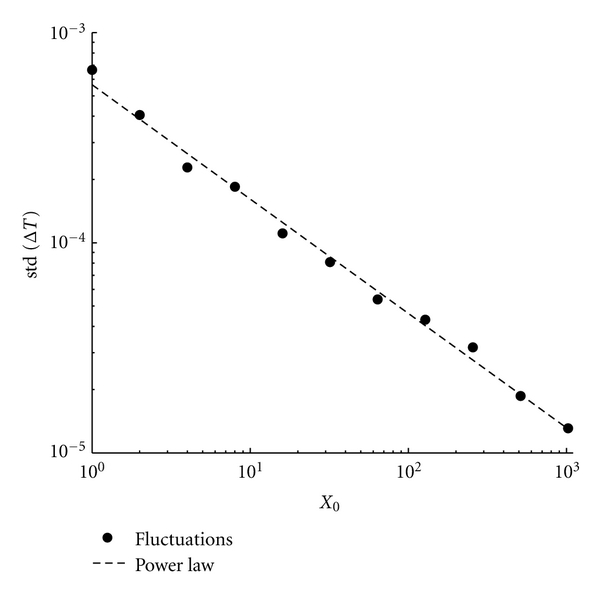
Fluctuation dependence on the initial conditions. We report the standard deviation of the protocell division time as a function of the initial amount of molecules *X*_0_ (•) and a linear best fit, whose slope is = −0.54 ± 0.03. Parameters are *X*(0) = 2^*n*^ with *n* = 0,…, 10, *C*(0) = 10*X*(0), *η* = 1, *α* = 1, *L*1 = 500, *P*1 = 600, *ρ* = 100, and *β* = 1.

To get a more complete understanding of the fluctuations dependence, we decided to measure them using the standard deviation of the protocell division time (after a sufficiently long transient phase). In Figure 7 we report the standard deviation of the division time Δ*T* as a function of the initial amount of molecules. As expected the fluctuations strength decreases rapidly as soon as the number of molecules increases and the relation can be very well approximated by a power law distribution with exponent −0.54 ± 0.03 (linear best fit).

### 5.2. Two Noninteracting Genetic Memory Molecules

A slightly more sophisticated model can be obtained by considering two linear non interacting GMMs. The system can be described by the following chemical reactions:

(33) $$\begin{array}{cc} & \text{channel}\;1,R_{1}:\;X_{1}+P_{1}\overset{\eta_{1}}{\to }2X_{1}\\ & \text{channel}\;2,R_{2}:\;X_{1}+L_{1}\overset{\alpha_{1}}{\to }X_{1}+C\\ & \text{channel}\;3,R_{3}:\;X_{2}+P_{2}\overset{\eta_{2}}{\to }2X_{2}\\ & \text{channel}\;4,R_{4}:\;X_{2}+L_{2}\overset{\alpha_{2}}{\to }X_{2}+C, \end{array}$$

where *P*_*i*_ and *L*_*i*_ are, respectively, precursors of the *i*th GMM, that is, nucleotide, and precursors of amphiphiles used by the *i*th GMM to build a *C* molecule.

**Figure 8:**
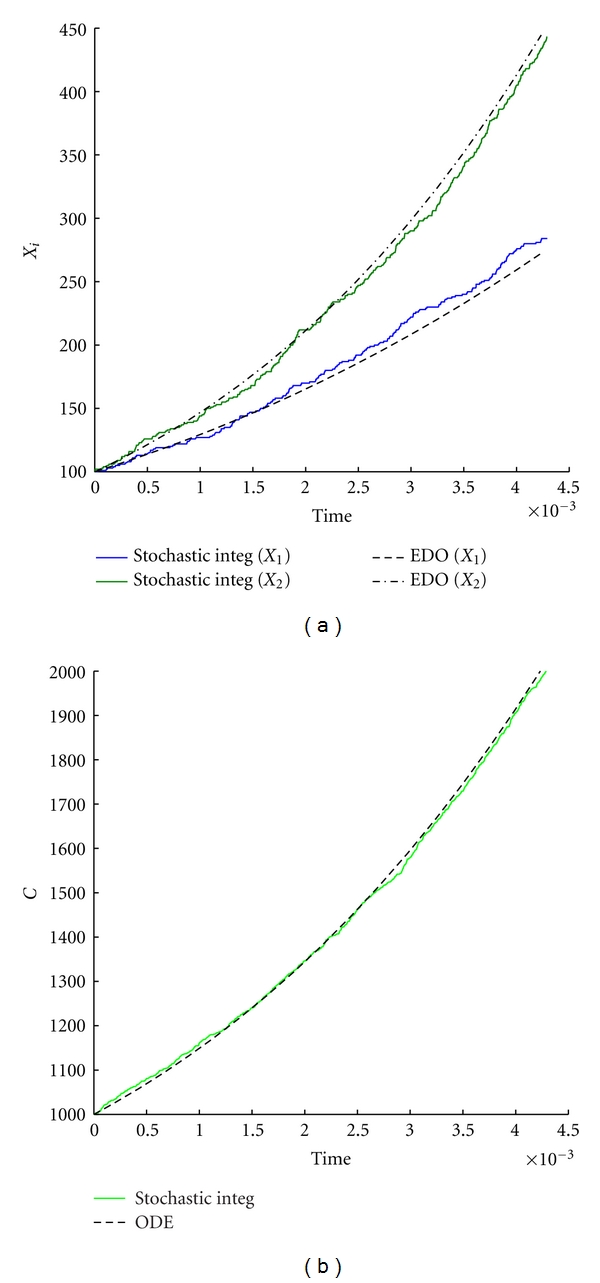
Stochastic versus ODE SRM protocell (3). Case of two GMMs, (a) the time evolution of the amount of GMM during a division cycle, (b) the time evolution of the amount of *C* molecules. Parameters are *η*_1_ = *η*_2_ = 1, *α*_1_ = *α*_2_ = 2, *L*1 = 500, *L*2 = 600, *P*1 = 600, *P*_2_ = 670, *X*_1_(0) = *X*_2_(0) = 100, *C*(0) = 1000, *ρ* = 200, and *β* = 2/3.

**Figure 9:**
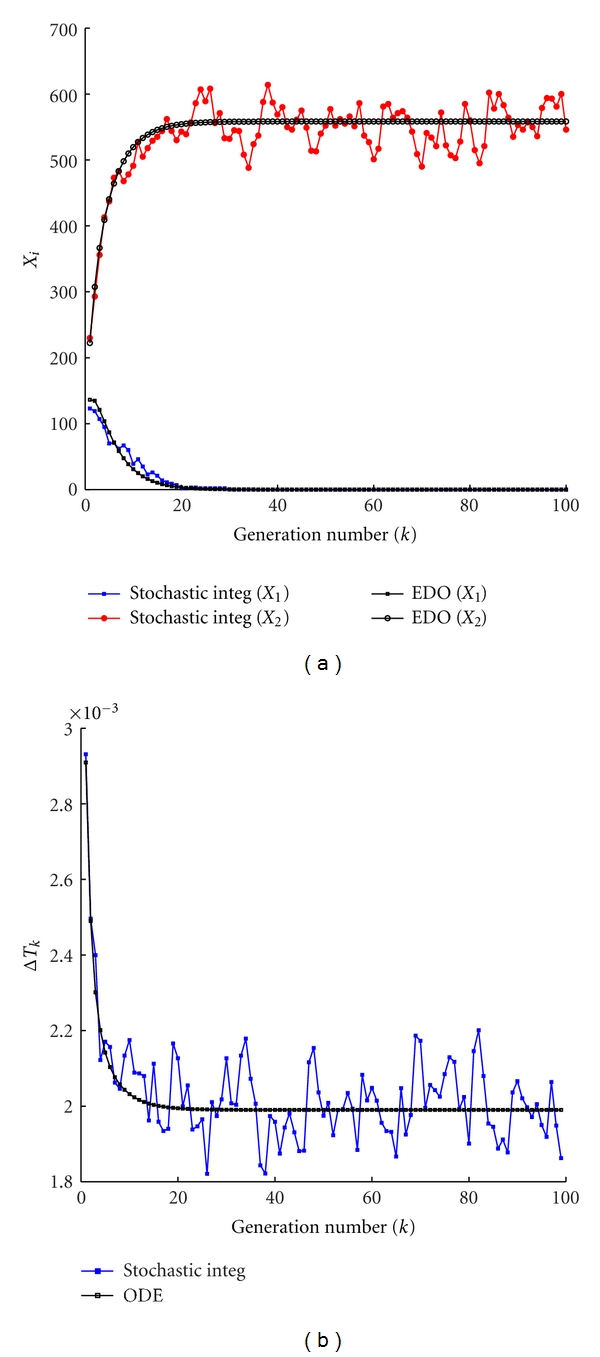
Stochastic versus ODE SRM protocell (3). Case of two GMMs, (a) the amount of GMM at the beginning of each division cycle, (b) the division time as a function of the number of elapsed divisions. Parameters are *η*_1_ = *η*_2_ = 1, *α*_1_ = *α*_2_ = 2, *L*1 = 500, *L*2 = 600, *P*1 = 600, *P*_2_ = 670, *X*_1_(0) = *X*_2_(0) = 100, *C*(0) = 1000, *ρ* = 200, and *β* = 2/3.

As previously done, we compare the stochastic and the deterministic models. Results are reported in Figure 8 and one can still observe that in presence of a large number of molecules the deterministic dynamics well approximates the stochastic model. On the other hand, the protocell division time exhibits large fluctuations around the deterministic value even in presence of quite large number of molecules (see Figure 9(b)).

The parameters have been set in such a way that only one GMM will survive according to the analytical theory for the deterministic model. One can observe that, despite the fluctuations, the same fate is obtained for the stochastic model (see Figure 9(b)).

**Figure 10:**
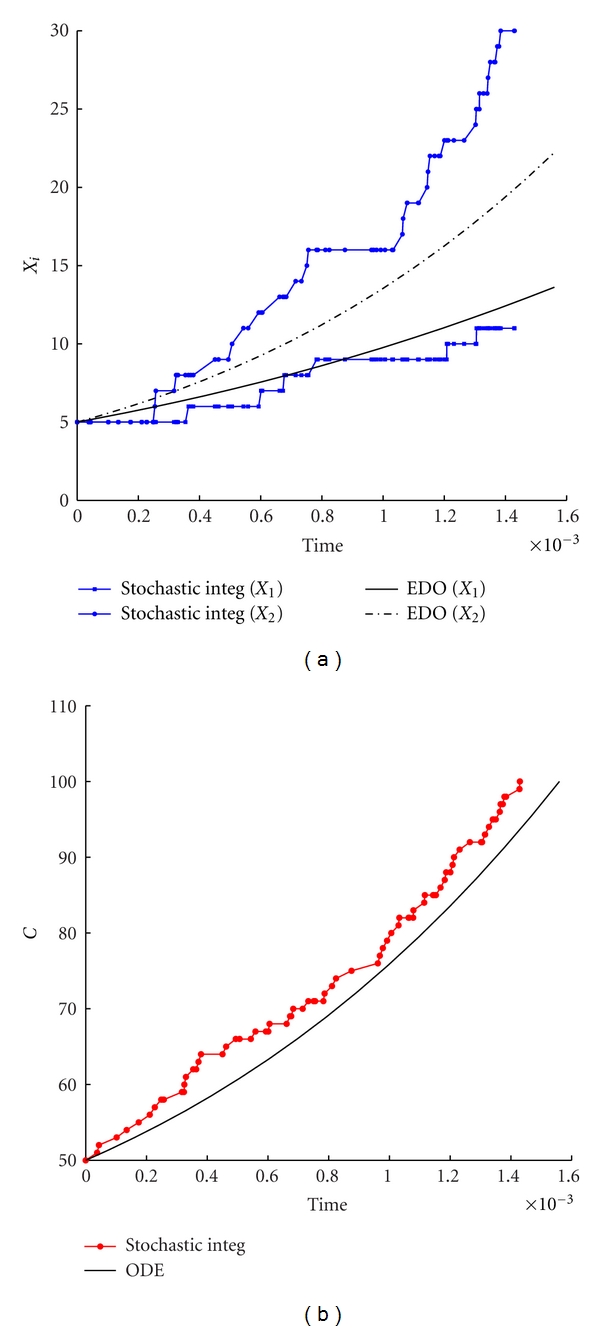
Stochastic versus ODE SRM protocell (3). Case of two GMMs, (a) the time evolution of the amount of GMM during a division cycle, (b) the time evolution of the amount of *C* molecules. Parameters are *η*_1_ = *η*_2_ = 1, *α*_1_ = *α*_2_ = 2, *L*1 = 500, *L*2 = 600, *P*1 = 450, *P*_2_ = 670, *X*_1_(0) = *X*_2_(0) = 5, *C*(0) = 50, *ρ* = 200, and *β* = 2/3.

**Figure 11:**
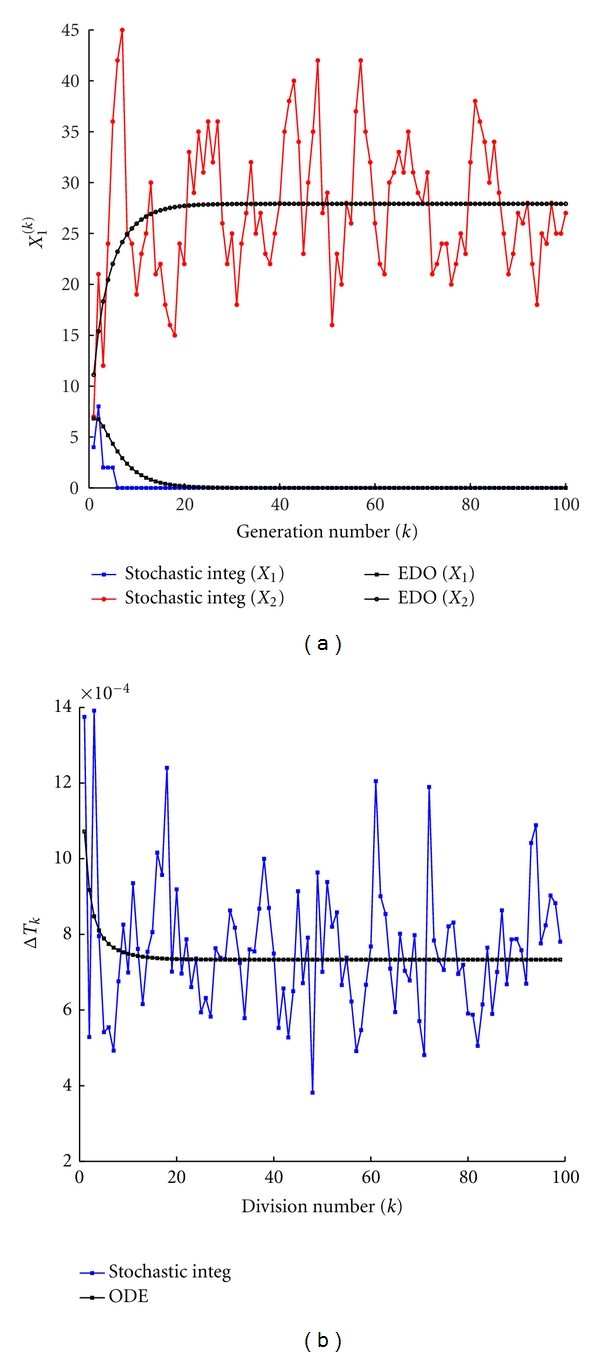
Stochastic versus ODE SRM protocell (3). Case of two GMMs, (a) the amount of GMM at the beginning of each division cycle, (b) the division time as a function of the number of elapsed divisions. Parameters are *η*_1_ = *η*_2_ = 1, *α*_1_ = *α*_2_ = 2, *L*1 = 500, *L*2 = 600, *P*1 = 450, *P*_2_ = 670, *X*_1_(0) = *X*_2_(0) = 5, *C*(0) = 50, *ρ* = 200, and *β* = 2/3.

Once we reduce the number of involved molecules, the stochastic fluctuations dramatically increase (see Figures 10 and 11).

**Figure 12:**
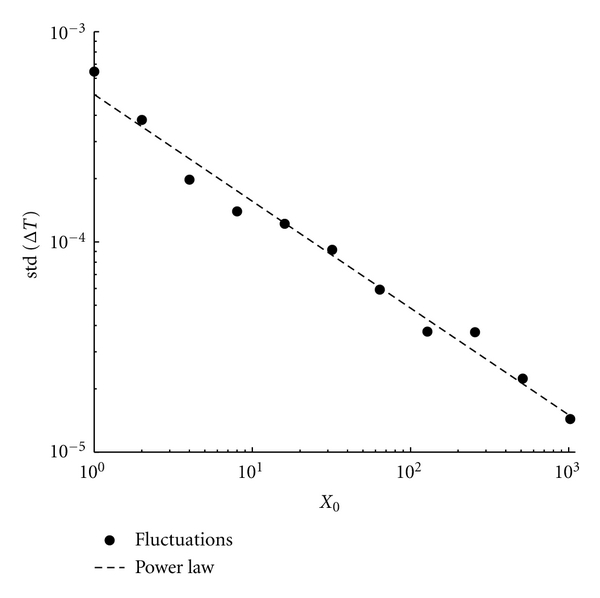
Fluctuation dependence on the initial conditions. We report the standard deviation of the protocell division time as a function of the initial amount of molecules *X*_*i*_(0), *i* = 1,2, (•) and a linear best fit, whose slope is = −0.51 ± 0.05. Parameters are *X*_1_(0) = *X*_2_(0) = 2^*n*^ with *n* = 0,…, 10, *C*(0) = 10*X*_1_(0), *η*_1_ = *η*_2_ = 1, *α*_1_ = *α*_2_ = 2, *L*1 = 500, *L*2 = 500, *P*1 = 500, *P*_2_ = 600, *ρ* = 100, and *β* = 1.

As in the case of only one GMM, when two non interacting linear GMMs are present the size of the stochastic fluctuations as a function of the initial number of molecules follows a power law distribution with exponent −0.51 ± 0.05 (linear best fit), see Figure 12: the fewer the molecules in the system are, the larger the fluctuations around the deterministic dynamics are.

**Figure 13:**
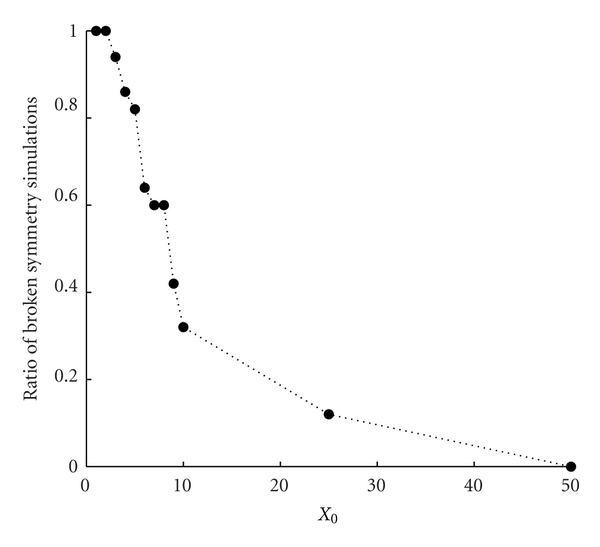
Symmetry breaking phenomenon. Each point denotes the fraction of runs exhibiting the symmetry breaking phenomenon, during 100 generations, over 50 identical replicas. Parameters are *X*_1_(0) = *X*_2_(0) = [1,2, 3,4, 5,6, 7,8, 9,10,25,50], *C*(0) = 10*X*, *η*_1_ = *η*_2_ = 1, *α*_1_ = *α*_2_ = 2, *L*1 = 500, *L*2 = 500, *P*1 = 600, *P*_2_ = 600, *ρ* = 100, and *β* = 1.

A new phenomenon arises in the case of two GMMs modeled by a stochastic process. There can be a *breaking of the symmetry* emerging in systems composed of two identical GMMs (i.e., equal kinetic constants, equal initial amounts, and availability of precursors) present with a few initial amounts of each one. Although adopting a deterministic approach the dynamics of the two replicators would be perfectly the same, a small fluctuation in the very first instants of the protocell evolution entails the dilution of one of the two replicators and thus a different fate for the protocell. Let us observe that the probability to have a large fluctuation is never zero, thus waiting for a sufficiently long time, a specie can always disappear from the system, thus giving rise to the breaking of the symmetry phenomenon. See Figure 13 where we report, as a function of the initial amount of molecules *X*_*i*_(0), *i* = 1,2, the proportion of simulations where the symmetry breaking has been observed repeating 50 times each simulation with the same set of parameters and initial conditions during 100 generations.

## 6. Conclusion

In this paper we presented a new stochastic integration algorithm based on the one introduced by Gillespie. Our contribution is devoted to the explicit introduction of the volume variation in the algorithm, which moreover is directly related to the amount of contained molecules, and thus it evolves in a self-consistent way.

This algorithm straightforwardly adapts to the study of the evolution of a protocell, simplified form of cells, where an ensemble of chemical reactions occurs in a varying volume, the volume of the protocell, that in turn increases because of the production of container molecules.

We presented several protocell models and we compare them with the analogous deterministic protocell models, namely, solved using the ODE. In this preliminary study, we emphasized the role of the fluctuations and their dependence on the initial amount of molecules. The dynamics is richer than the deterministic one and thus it is worth studying, in particular we deserve to future investigations the case where the interactions among the molecules can be modeled by a linear system, whose interaction matrix is not diagonal; the off diagonal terms representing the cross-catalysis. Also the case of nonlinear interactions will be deferred to a forthcoming paper. Also the study of the emergence of time-periodic patterns due to the fluctuations, will be analyzed. An analytical treatment of the latter case could be possible using some recent technics developed by [15, 16], see also [17] where the space is also taken into account.
